# Supplementary material for: Non-human primate LIBRA-Seq accelerates neutralizing antibody discovery in RM vaccinated against HIV-1
Source: PLoS Pathog. 2026 Jul 28;22(7):e1013831. doi: 10.1371/journal.ppat.1013831 (PMC13411919; doi:10.1371/journal.ppat.1013831)
Supplement: S1 Text — Fig A in S1 Text. Construction of LIBRA-Seq compatible BG505 SOSIP probes. (A1) Staining pmel splenocytes for Gp100-specific T cells with Db-Gp100-tetramers made from various streptavidin. (A2) Gel electrophoresis of StvC-AF-DNA conjugates stained with Coommisie Blue (left) and SYBR DNA Gold (right). Fig B in S1 Text. Validation of LIBRA-Seq compatible BG505 SOSIP probes in vitro. (B1) Flow cytometry gating strategy for the identification of VRC01 or RA.1 expressing Ramos B cells to DNA-barcoded, fluorescently labeled BG505 SOSIP. Cells were gated on FSC and SSC characteristic of singlets, Ramos cells, live cells, CD19 + , IgM + , BG505-AF647 + . (B2) Fluorescence-minus-one plots for RA.1 (top) or VRC01 (bottom) expressing B cells. Cells were stained without BG505-AF647+ to assess background fluorescence in the AF647 channel. (B3) Histogram displaying the number of antigen barcode reads associated with 10x captured VRC01 (blue) or Ra.1 (orange) Ramos B cells. Fig C in S1 Text. Validation of RA.1 Ramos Cell line CDR3 usage. Distribution of heavy and light chain CDR3 usage among 10x captured VRC01 (C1) and Ra.1 (C2) Ramos B cells. Higher percentages denoted in red and lower in light blue. Canonical VRC01 and Ra.1 CDRH3 and CDRL3 are bolded. Fig D in S1 Text. Validation of LIBRA-Seq compatible BG505 SOSIP probes in vivo. Feature scatter plots highlighting the raw (D1) and normalized (D2) read counts for LIBRA-Seq barcodes and show each combination of the biotin control and two BG505 SOSIP baits. The dotted lines represent the thresholds for antigen barcodes that were chosen empirically - 97th percentile for biotin negative control and 3rd percentile for the two BG505 SOSIP barcodes. Each dot represents a unique cell. Fig E in S1 Text. Isolation of BG505 SOSIP specific memory B cells from vaccinated RM. (E1) Fluorescence activated cell sorting gating strategy for the isolation of antigen specific memory B cells from cryopreserved PBMCs from vaccinated RM collected at ti [file ppat.1013831.s001.docx]

Supplementary Materials

***LIST OF SUPPLEMENTARY MATERIALS***

Fig. A to G

Data Files S1-S4
