# Supplementary material for: Non-human primate LIBRA-Seq accelerates neutralizing antibody discovery in RM vaccinated against HIV-1
Source: PLoS Pathog. 2026 Jul 28;22(7):e1013831. doi: 10.1371/journal.ppat.1013831 (PMC13411919; doi:10.1371/journal.ppat.1013831)
Supplement: Attachment — Submitted filename: Response to Reviewers.docx [file ppat.1013831.s007.docx]

**Responses to Reviewer Comments:**

**Reviewer #1:** The manuscript by Edwards et al. describes the adaptation of LIBRA-seq to characterize antibody responses in rhesus macaques. The authors developed DNA-barcoded BG505 SOSIP probes for sorting antigen-specific B cells, 10X capture, and single-cell RNA sequencing. They validated this approach using engineered cell lines and then applied it to identify antigen-specific memory B cells from six vaccinated animals. This approach recovered four previously identified neutralizing antibodies from animal RUp16, while identifying six additional neutralizing clones across the cohort. High-throughput analysis revealed patterns of clonal expansion and shared genetic features across animals. The LIBRA-seq scores appear useful for prioritizing antibody candidates for functional testing. However, the manuscript lacks a direct comparison between LIBRA-seq and conventional antibody cloning methods, as well as a discussion of LIBRA-seq’s limitations. Most notably, while the authors assert that LIBRA-seq "accelerates" antibody discovery, they provide no quantitative comparisons of cost, time, or workflow efficiency relative to conventional approaches. Additionally, the identification of a potent neutralizing antibody (RUp16_158_TriplePositive) that would have been excluded based on their filtering strategy, which requires biotin-bait negativity, raises concerns about the analytical pipeline that are inadequately addressed. With revisions addressing these comparisons and technical limitations, this work would represent a valuable methodological contribution to preclinical non-human primate studies.

**Reviewer #2:** In this study, Edwards et al apply the LIBRA-seq platform to memory B cells from rhesus macaques that received an experimental HIV vaccine, enabling immunogen-specific between IgH/L sequences to be linked with their antigen specificity. They report findings from a total of 6 vaccinated animals, one of which was previously described to have mounted a particularly potent response. The authors validate the performance of their approach by analyzing the recovery of previously-characterized clones, and also by expressing newly-identified antibodies, which together indicate good sensitivity and specificity. Overall, the study is soundly-designed, well-communicated and its conclusions are supported by the data shown.

In light of the prior studies by these authors on the same types of antibodies from the same animals, and reaching a number of the same scientific conclusions, I note that the main import of the work is as a methodological advance. However, these relevant citations are included and I think this methodological focus is frankly communicated and discussed.

**Reviewer #3:** Edwards, et.al., describe the adaptation and a proof-of-concept dataset demonstrating LIBRA-seq identification of binding and neutralizing antibodies targeting BG505 SOSIP in the rhesus macaque model of HIV/SIV vaccination. The team demonstrates that the adapted method is robust, reproduces prior results, and increases screening throughput for the identification and characterization of antigen-specific monoclonal antibodies. These results represent a discernable improvement over more classical, plate-based methods, and will allow more robust evaluation of humoral responses in the context of pre-clinical studies with non-human primates. This reviewer would like to commend the authors on the extremely thorough description of methodological details, which will surely encourage others to build from these findings. Overall, the paper describes a new approach that will surely increase our understanding of antibody responses during pre-clinical vaccination studies and, with relatively minor updates, should be acceptable for publication in PLoS Pathogens.

We thank the reviewers for their kind words and constructive feedback.

Major Concerns

Reviewer #1

1. The authors assert that LIBRA-seq "accelerates" neutralizing antibody discovery in rhesus macaques. However, they provide no quantitative data to support this claim. The manuscript should include explicit comparisons of (i) hit rates for neutralizing antibody identification between LIBRA-seq and conventional approaches and (ii) estimates of the time required from sample collection to antibody characterization for each approach. Given that the authors previously characterized RUp16 using conventional methods, these comparative data should be readily available. Without such quantitative support, claims of improved efficiency cannot be substantiated.

We agree with the reviewer that our study was lacking an empirical comparison of LIBRA-Seq with conventional methodology, and also agree that this omission undermined our conclusion that LIBRA-Seq sped up antibody discovery. Based on their suggestion, we have used the records from our (T. Charles, C. Derdeyn) prior study (Charles et al. PLoS Pathogens) in which we identified neutralizing antibodies from the RUP16 animal using conventional methods to directly compare against the LIBRA-Seq analysis on the same animal reported in the current manuscript. We have summarized the comparison as a new display item (Table 2) and added a paragraph at in the Results (see below). Briefly, the hit rate for antibodies with neutralizing activity using conventional methods from RUP16 was 4/117 (4%); for LIBRA-Seq it was 5/5 for dual positive mAbs (100%) but was biased as we had prior knowledge of neutralizing lineages. For non-RUP16 animals, the overall hit rate was 4/24 (17%). Based on this, a conservative estimate of the increase in hit rate is 4x; given that hit rate in conventional animals would likely be less than the rate of 4% seen in RUP16. In terms of time, we were able to generate sequence data in 2 to 4 weeks after our sorting experiment (times vary for each animal). In comparison, the time to generate sequences after sorting using conventional methods was 6 months (for BG505 trimer-sorted cells) and an additional 3 months for BG505 gp120 monomer sorted cells; we have summarized this information in the newly added Table 2. Overall, we feel that inclusion of this comparison strengthens our contention that nhp-LIBRA-Seq can greatly reduce the time and labor needed to discover neutralizing antibodies. We thank the Reviewer for their careful reading of our manuscript and suggestion of this analysis, which we feel has improved the study.

2. The observation that 50% (2/4) of antibodies with high biotin-bait LIBRA-seq scores bound BG505 SOSIP by ELISA, with Rup16_158_TriplePositive exhibiting potent neutralizing activity, indicates that the biotin-negative filtering strategy may misclassify antigen-specific clones. The authors should quantify how many cells were excluded based on biotin-positivity across the entire cohort and determine whether these excluded cells cluster with double-positive for BG505 and negative for biotin (as observed for RUp16_158_TriplePositive and its dual-positive clonal relatives). They should also discuss the implications of these findings and recommend filtering options for future studies implementing LIBRA-seq.

We thank the reviewer for this insightful comment and agree that the identification of RUp16_158_TriplePositive highlights an important limitation of the filtering strategy used in the original analysis. In the revised manuscript, we now explicitly acknowledge that strict binary exclusion of biotin-positive cells can misclassify bona fide antigen-specific clones.

To address this concern, we re-analyzed antigen-barcode patterns across the full cohort. This analysis showed that the large majority of recovered cells fell within the expected dual-positive, biotin-negative compartment, but also identified a small triple-positive population. Importantly, a substantial fraction of these triple-positive cells were clonally linked to accepted BG505-specific cells when dual- and triple-positive cells were analyzed together. Notably, all shared triple-positive clone membership was observed in RUp16, where triple-positive cells were found within clones also represented in the dual-positive compartment, including the RUp16_158 lineage.

We now summarize these cohort-level findings in the Results and use them in the Discussion to clarify that, while the negative bait remains useful for reducing nonspecific signal, hard exclusion of all biotin-positive events may be overly stringent and can reduce sensitivity in certain cases. We therefore recommend that future implementations of this workflow treat negative-bait signal as a quantitative quality-control feature rather than a strict binary exclusion criterion, particularly when the goal is rare-lineage discovery.

Reviewer #2: (No Response)

Reviewer #3:

- Antibody clonal assignments were made by identifying shared V and J genes, and shared CDR3 length, alongside CDR3 identity >70% at the nucleotide level. This seems quite a low identity threshold for a clonal grouping. Further explanation as to how and why these parameters were set for identifying members of the same clonal group would strengthen the overall narrative.

We thank the reviewer for this thoughtful critique and agree that the original threshold used for within-animal clonal assignment was too permissive. In the revised manuscript, we have repeated our intra-animal clonal analysis using a more stringent 85% CDR3 nucleotide identity threshold, together with shared V gene usage, shared J gene usage, and shared CDR3 length. However, for the identification of public clones across animals, we retained the 70% CDR3 identity threshold, as this is more consistent with thresholds used in recent studies of convergent or public antibody responses in the field (1, 2). We have updated the Methods, Results, and relevant figure legends to clarify the rationale for these parameters and to reflect the revised intra-animal clonal analyses, and we now cite the appropriate supporting references.

- Figure 4 and associated text demonstrate comparative analyses between the bnAbs identified by LIBRA-seq versus prior studies using more conventional methods in animal RUp16. Approximately 50% (24/48) of the identified heavy chains and 90% (44/48) of light chains agreed with prior analyses. Although the agreement on light chains is clear, the authors do not comment on the lack of overlap of heavy chains. Further discussion on whether this may represent sampling bias or due to a different time point examined would be helpful to agree with the authors conclusions that this constitutes clear orthogonal validation of the LIBRA-seq approach.

We thank the reviewer for this thoughtful comment and agree that the incomplete overlap in recovered heavy chains between the LIBRA-Seq data set and the prior conventional analysis of RUp16 warrants further comment. In the original manuscript our intent was to emphasize that LIBRA-Seq recovered the previously identified nAb clonal lineages from this animal, which we view as the strongest validation metric. We note that the lower overlap in heavy chain likely reflects several factors. First- the inherent diversity of heavy chains within a repertoire is greater which would decrease the frequency of shared sequence recovery. Second, the definition used for clonal overlap included 85% nt identity across the CDRH3; at this stringency, it is common to see related lineages fall below this threshold. Third, and perhaps most importantly, as the reviewer notes, the difference in time-points used for the each of the methods had a strong effect on the number of unique clonotypes available for sampling. In the conventional methodology, we sampled RUP16 at week 44, 4 weeks after the third Env protein trimer vaccination; for LIBRA-Seq we sorted from PBMCs taken at weeks 87 and 90, after the fourth vaccine at week 80. In a prior study, we observed that the number of clonotypes-per-recovered Env-specific B cells decreased after additional protein Env vaccinations{Mopuri 2023}, reflecting the expansion of individual clonal lineages and reduction of overall repertoire diversity. Importantly, we were to assess if this narrowing of the repertoire after protein boosts occurred in RUP16, using data from a companion manuscript (currently in revision at PLoS Pathogens as Myers et al.). As shown below, we observed that at wks 48 and 49, after the 3^rd^ vaccine (week 40), expanded clones comprised <25% of all clonotypes; in contrast, at week 81, 83 and 97, the repertoire was substantially more oligoclonal, with large clonotypes comprising up to 50% of the repertoire. Collectively, these demonstrate that there is less diversity of the BG505-specific repertoire after the fourth vaccination; or put another way, less overall clonotypes available for sampling. Combined with the inherent higher diversity of the heavy-chain compared to Iight chains, we feel the different time-points sampled was likely the largest contributor to the lower rate of congruency observed for heavy chains. Lastly, we also considered whether technical biases inherent to LIBRA-Seq may explain the disparity in recovery levels. Of note, in prior studies (Mopuri et al. PLoS Pathogens) we have observed a significantly lower levels of cells with productive H chains relative to K/L chains, attributed to the longer length of the amplicon, and fewer cells that pass sequencing coverage thresholds. However, in this study, we only used LIBRA-Seq recovered cells with both IgH and IgK/L sequences meeting QC thresholds. Other than the bioinformatic thresholding issue we have described above, we do not feel that technical biases played a major role in the disparity between our light chain and heavy chain recovery rates.

We have now expanded our discussion of the *in vivo* benchmarking results to specifically highlight the discrepancy in sequence recovery. These revisions clarify our interpretation by distinguishing between the main orthogonal validation metric (nAb sequence recovery) and incomplete sequence-level overlap across the broader heavy chain repertoire, and place it in the context of the differing sampling time-points used. We think this suggestion addition improves the quality of the manuscript overall, and appreciate the attention to detail by the reviewer.

**(A) Clonal diversity of BG505-specific MBCs in animal RUP16 across longitudinal time points.** Bar plot showing the proportional abundance of clonotypes that appear in the population between 0.1 and 2% (small, purple), 2% and 10% (medium, pink) or 10% and 30% (large, orange) from paired VDJ/VJ sequences recovered from BG505-specific cells. BG505.SOSIP trimers were administered at week 16, week 24, week 40 and week 80. Data sourced from Myers et al. *in revision*, PLoS Pathogens.

- The final Results section defines repertoire features associated with those monoclonal Abs identified by the LIBRA-seq method, including the public usage of IGHV4-79. This is a great example of how this type of method could identify new germline alleles to be targeted by rationally designed immunogens using reverse vaccinology. It was unclear whether the additional animals examined (beyond Rup16) had been characterized using more conventional methods. If the data are available, it would considerably strengthen the overall conclusions to perform similar analyses with “conventional” data. In the animals (or, if not available, if just in Rup16) did the authors see the use of IGHV4-79? Did they see high levels of SHM? In general, these types of broader analyses between standard and LIBRA-seq methods would assist in alleviating any concerns regarding LIBRA-seq-specific bias in repertoire capture and characterization.

We thank the reviewer for this thoughtful comment and agree that broader conventional comparison would strengthen the interpretation of recurrent repertoire features identified by LIBRA-seq. At present, directly comparable conventional monoclonal datasets are available only for RUp16 and not for the full cohort analyzed here. We have therefore revised the text to narrow the scope of these conclusions.

In the revised manuscript, we now frame recurrent features such as IGHV4-79 usage and SHM patterns as descriptive characteristics of the LIBRA-seq-captured antigen-specific repertoire in this cohort, rather than definitive evidence that these features are uniquely enriched across platforms or universally representative of the total vaccine-elicited repertoire. We also add discussion that broader cross-platform validation of these repertoire-level observations in additional animals will be an important future direction.

This change better reflects the available data while preserving the central point that LIBRA-seq can reveal potentially important recurrent immunogenetic features for follow-up.

- This adapted LIBRA-seq method identified both binding and neutralizing antibodies. The most important concern for the vaccine field is to focus on the identification of bnAbs, as binding antibodies are developed at high titers in most infected individuals. It would be beneficial for the authors to discuss possible future improvements to the assays or analyses parameters that may enhance the discernment between binding and neutralizing antibody identification.

We agree with the reviewer that antigen binding alone is insufficient to distinguish neutralizing from non-neutralizing antibodies and that this limitation warrants clearer discussion. We have now expanded the Discussion to address how future iterations of this workflow could improve functional discrimination.

Specifically, we discuss that future studies could incorporate multiplexed panels of Env probes representing different epitope states, glycan presentations, or conformationally selective baits, thereby enabling more informative binding signatures than those generated by a single antigen probe. We also note that LIBRA-seq is best viewed as complementary to downstream functional screening workflows, rather than a replacement for them, and that integration with functional platforms such as antibody expression and neutralization testing will remain essential for identifying broadly neutralizing antibodies with confidence.

- Data sharing remains unclear, as GEO accessions are “TBD” and git-hub documentation “will be made available”. These availabilities need to be guaranteed and made available to reviewers prior to acceptance.

We have now updated the manuscript to provide the GEO accession number (GSE329552) for the deposited sequencing data and have added the analysis scripts and supporting documentation to our GitHub: <https://github.com/BosingerLab/U24_Antigen_barcoding>.

To review GEO accession GSE329552:
Go to [https://nam11.safelinks.protection.outlook.com/?url=https%3A%2F%2Fwww.ncbi.nlm.nih.gov%2Fgeo%2Fquery%2Facc.cgi%3Facc%3DGSE329552&data=05%7C02%7Cgktharp%40emory.edu%7C83901f1a6b5b4b22fe6f08dea63000cf%7Ce004fb9cb0a4424fbcd0322606d5df38%7C0%7C0%7C639130922282279782%7CUnknown%7CTWFpbGZsb3d8eyJFbXB0eU1hcGkiOnRydWUsIlYiOiIwLjAuMDAwMCIsIlAiOiJXaW4zMiIsIkFOIjoiTWFpbCIsIldUIjoyfQ%3D%3D%7C0%7C%7C%7C&sdata=klBtFKO4oJu88TnPntKNZkI4Ft0nKEsc%2BFYhYnEOum8%3D&reserved=0](https://nam11.safelinks.protection.outlook.com/?url=https%3A%2F%2Fwww.ncbi.nlm.nih.gov%2Fgeo%2Fquery%2Facc.cgi%3Facc%3DGSE329552&data=05%7C02%7Cchristopher.edwards%40emory.edu%7C60881fea400e41c63e9008dea6304a37%7Ce004fb9cb0a4424fbcd0322606d5df38%7C0%7C0%7C639130923505599224%7CUnknown%7CTWFpbGZsb3d8eyJFbXB0eU1hcGkiOnRydWUsIlYiOiIwLjAuMDAwMCIsIlAiOiJXaW4zMiIsIkFOIjoiTWFpbCIsIldUIjoyfQ%3D%3D%7C0%7C%7C%7C&sdata=ud6Gp1hGt%2FOOxJFWHQHQxLR%2FhtOad06IYxV8XC0gyG8%3D&reserved=0)
Enter token whwrwcagndelher into the box

**Minor Issues:**

Reviewer #1:

 1. The authors should include more discussion of the technical challenges of implementing LIBRA-seq and the approach's limitations, including practical considerations for those considering LIBRA-seq, the technical expertise required, and the computational resources. This information will help readers assess the feasibility of implementing LIBRA-seq.

We agree with the reviewer that the original Discussion did not adequately describe the practical challenges and limitations of implementing this approach. We have now expanded the Discussion to include a more explicit overview of the technical and analytical requirements of the NHP-adapted LIBRA-seq workflow and have included some potential hurdles of the technique in figure 2.

Specifically, we now discuss that this approach requires: i) custom generation and validation of DNA-barcoded antigen probes, ii) high-quality flow cytometric enrichment of antigen-specific cells compatible with 10X Genomics capture, iii) integration of feature-barcode, V(D)J, and gene-expression data streams, iv) empirically selected barcode thresholds that may require dataset-specific optimization, and v) downstream cloning and functional validation to distinguish binding from neutralizing antibodies. We believe these additions provide a more balanced view of the method’s strengths and practical limitations.

2. HVV should be defined the first time it is used in the manuscript.

HVV has now been defined at its first instance in the manuscript.

Reviewer #2:

Given that only a single antigen is being used, the contribution of the DNA-barcode on the antigen probes is somewhat unclear: almost all of the discrimination appears to have been done by the fluorescent sorting (eg see Fig 3). How much would the sensitivity / specificity of recovering immunogen-specific IgH/Ls actually have dropped if there was no DNA barcode quantification available? In addition to excluding what appear to be a small number of fluorescent false positives, perhaps the main value of the barcode is allowing a quantitative estimation of binding signal strength / reliability? In that vein, it would have been helpful to see a more thorough and quantitative analysis of sensitivity / specific across the LSS range, and/or some further discussion on this point.

We thank the reviewer for this thoughtful comment and agree that, in a single-antigen proof-of-concept implementation, the added value of the DNA barcode requires clearer explanation. In the revised manuscript, we have clarified that in this study the DNA barcode served three main purposes: i) as an orthogonal confirmation of probe-associated signal after fluorescent sorting, ii) as a quantitative measure to rank candidate cells by antigen-associated barcode signal, and iii) as a scalable framework for future multiplexed antigen panels, even though the present study used only one antigen plus controls.

We also agree that the original manuscript did not sufficiently communicate how barcode signal strength related to candidate prioritization. To address this, we have expanded the Discussion to state that, in the current study, the barcode’s value was less in replacing FACS discrimination and more in refining specificity assessment, prioritizing candidate sequences, and establishing a platform for future multi-antigen applications.

We have intentionally avoided overstating the barcode’s contribution in this single-antigen context and now describe it more precisely as a quantitative and orthogonal prioritization feature.

– The titration in Figure 2 would have benefitted from replicates, or an explanation of why none were performed.

An updated Figure 2 including 3 replicates has been included in the manuscript.

– Fig 6: "D" missing from legend.

The figure legend has been updated.

Reviewer #3:

In general, the paper was well written and clear. There were a number of typos and minor comments listed here to assist in the readability of the manuscript once it is published:

- Line 269: there are words missing at the end of this sentence, “…0.022-0.039 in IgK, and (0.025-0.044).
- Line 283: I believe that this wording should be adjusted to, “high resolution, higher throughput manner…” to more accurately represent the data presented.
- Lines 289-291, “In addition to recovering the previously identified neutralizing clones, we were able to identify shared gene usage and clonotypes across multiple variants.” While not untrue, this statement felt like a bit of an overreach, as the authors did not identify an exact overlap between methods. This reviewer suggests rewording to “…was able to identify partial shared gene usage…” to better temper the results.
- Lines 428 and 440. Please use the correct symbol for degrees.
- Line 447, this sentence starts with, “(AMQAX1000) Appropriate volume…” Clearly something was lost in editing, please correct.
- Line 452-453, Poor grammar and articles are missing; please review and correct.
- Throughout manuscript – the Ramos cell line is referred to as “ramos” and “Ramos”. Please decide regarding capitalization and be consistent throughout the manuscript.
- Lines 533-534, it is unclear how and what was “combined at 1 to 1 overnight…”. Were streptavidin-AF and oligonucleotide mixed at 1:1 based on vol:vol, or mass:mass? Clarify methods here to increase reproducibility.
- Multiple times the term “x” was used as shorthand for replicates. Please expand. For example, line 551, “Splenocytes were washed 3x with FACS buffer…” should be “Splenocytes were washed three times with FACS buffer...”.
- Single-cell RNA-seq using 10X Genomics equipment and reagents was critical for this study. However, these supplies are referred to repeatedly as either “10x” or “10X”. Please use the proper “10X Genomics” and be consistent throughout draft.
- Lines 626-636, this section appears to be completely duplicated from the prior section (lines 590-601) and can be removed.
- The “Libra-seq Scoring” methods section (lines 637-649) also seems almost word-for-word duplicated from an earlier methods section (lines 602-612). Please rework to limit redundancy.

The suggested changes have been made in the manuscript.

References

1.	Waury K, Lelieveld S, Abeln S, Ham H-Jvd. Comparison of sequence- and structure-based antibody clustering approaches on simulated repertoire sequencing data. PLoS Comput Biol. 2025;21(5):e1013057.

2.	Abu-Shmais AA, Vukovich MJ, Wasdin PT, Suresh YP, Marinov TM, Rush SA, et al. Antibody sequence determinants of viral antigen specificity. Mbio. 2024;15(10):e01560–24.
