# Supplementary material for: Non-human primate LIBRA-Seq accelerates neutralizing antibody discovery in RM vaccinated against HIV-1
Source: PLoS Pathog. 2026 Jul 28;22(7):e1013831. doi: 10.1371/journal.ppat.1013831 (PMC13411919; doi:10.1371/journal.ppat.1013831)
Supplement: Peer review history

# Peer review: Decision Letter 0

**DOI:** 10.1371/journal.ppat.1013831.r001

**Authors:** Susan R. Ross, David T. Evans

3 Mar 2026

-->PPATHOGENS-D-25-03219

Non-human primate LIBRA-Seq accelerates neutralizing antibody discovery in RM vaccinated against HIV-1

PLOS Pathogens

Dear Dr. Bosinger,

Thank you for submitting your manuscript to PLOS Pathogens. After careful consideration, we feel that it has merit but does not fully meet PLOS Pathogens's publication criteria as it currently stands. Therefore, we invite you to submit a revised version of the manuscript that addresses the points raised during the review process.

Please submit your revised manuscript by May 02 2026 11:59PM. If you will need more time than this to complete your revisions, please reply to this message or contact the journal office at plospathogens@plos.org. When you're ready to submit your revision, log on to https://www.editorialmanager.com/ppathogens/ and select the 'Submissions Needing Revision' folder to locate your manuscript file.

Please include the following items when submitting your revised manuscript:

* A letter that responds to each point raised by the editor and reviewer(s). You should upload this letter as a separate file labeled 'Response to Reviewers'. This file does not need to include responses to any formatting updates and technical items listed in the 'Journal Requirements' section below.

* A marked-up copy of your manuscript that highlights changes made to the original version. You should upload this as a separate file labeled 'Revised Manuscript with Track Changes'.

* An unmarked version of your revised paper without tracked changes. You should upload this as a separate file labeled 'Manuscript'.

If you would like to make changes to your financial disclosure, competing interests statement, or data availability statement, please make these updates within the submission form at the time of resubmission. Guidelines for resubmitting your figure files are available below the reviewer comments at the end of this letter.

We look forward to receiving your revised manuscript.

Kind regards,

David T. Evans

Academic Editor

PLOS Pathogens

Susan Ross

Section Editor

PLOS Pathogens

Sumita Bhaduri-McIntosh

Editor-in-Chief

PLOS Pathogens

orcid.org/0000-0003-2946-9497

Michael Malim

Editor-in-Chief

PLOS Pathogens

orcid.org/0000-0002-7699-2064

**Journal Requirements:**

1) Please ensure that the CRediT author contributions listed for every co-author are completed accurately and in full.

At this stage, the following Authors/Authors require contributions: Amanda Metz. Please ensure that the full contributions of each author are acknowledged in the "Add/Edit/Remove Authors" section of our submission form.

The list of CRediT author contributions may be found here: https://journals.plos.org/plospathogens/s/authorship#loc-author-contributions

2) We ask that a manuscript source file is provided at Revision. Please upload your manuscript file as a .doc, .docx, .rtf or .tex. If you are providing a .tex file, please upload it under the item type u2018LaTeX Source Fileu2019 and leave your .pdf version as the item type u2018Manuscriptu2019.

3) We do not publish any copyright or trademark symbols that usually accompany proprietary names, eg ©,  ®, or TM  (e.g. next to drug or reagent names). Therefore please remove all instances of trademark/copyright symbols throughout the text, including:

- ® on page: 31

- TM on page: 34.

4) Please upload all main figures as separate Figure files in .tif or .eps format. For more information about how to convert and format your figure files please see our guidelines:

https://journals.plos.org/plospathogens/s/figures

5) Some material included in your submission may be copyrighted. According to PLOSu2019s copyright policy, authors who use figures or other material (e.g., graphics, clipart, maps) from another author or copyright holder must demonstrate or obtain permission to publish this material under the Creative Commons Attribution 4.0 International (CC BY 4.0) License used by PLOS journals. Please closely review the details of PLOSu2019s copyright requirements here: PLOS Licenses and Copyright. If you need to request permissions from a copyright holder, you may use PLOS's Copyright Content Permission form.

Please respond directly to this email and provide any known details concerning your material's license terms and permissions required for reuse, even if you have not yet obtained copyright permissions or are unsure of your material's copyright compatibility. Once you have responded and addressed all other outstanding technical requirements, you may resubmit your manuscript within Editorial Manager.

Potential Copyright Issues:

i) Figure 1B. Please confirm whether you drew the images / clip-art within the figure panels by hand. If you did not draw the images, please provide (a) a link to the source of the images or icons and their license / terms of use; or (b) written permission from the copyright holder to publish the images or icons under our CC BY 4.0 license. Alternatively, you may replace the images with open source alternatives. See these open source resources you may use to replace images / clip-art:

- https://commons.wikimedia.org

- https://openclipart.org/.

ii) Figures 1A, 2A,3A, and 3B. We noted that the figures are created through BioRender. Please confirm that you hold a Premium account and provide a pdf copy of the CC BY 4.0 Licence as provided by BioRender. For instructions on how to generate a CC BY 4.0 license for your figure, please see the guidelines here: https://help.biorender.com/hc/en-gb/articles/21282341238045-Publishing-in-open-access-resources.

If you are using the free assets from BioRender, we are unable to publish these images as they are licenced under a stricter licence than CC BY 4.0. In this case we ask you to remove the BioRender images and replace them with open source alternatives.

See these open source resources you may use to replace images / clip-art:

- https://bioart.niaid.nih.gov/

- https://bioicons.com/

- https://healthicons.org/

- https://scidraw.io/

- https://reactome.org/icon-lib

- https://www.phylopic.org/images

- https://journals.plos.org/plosbiology/article?id=10.1371/journal.pbio.3002395

6) Thank you for stating "Data tables for expression counts for single-cell RNA-seq for memory B cells are are deposited in NCBI’s Gene Expression Omnibus and are accessible through the Gene Expression Omnibus (GEO) under accession number (TBD)." Please provide us with the accession number of the dataset.

7) Thank you for indicating that "Custom scripts and supporting documentation on the RNA-seq analyses will be made available at "https://github.com/BosingerLab/NHP_LIBRA-Seq" We strongly recommend all authors deposit their data before acceptance, as the process can be lengthy and hold up publication timelines. Please note that, though access restrictions are acceptable now, your entire minimal dataset will need to be made freely accessible if your manuscript is accepted for publication.

8) Please amend your detailed Financial Disclosure statement. This is published with the article. It must therefore be completed in full sentences and contain the exact wording you wish to be published.

1) If the funders had no role in your study, please state: "The funders had no role in study design, data collection and analysis, decision to publish, or preparation of the manuscript."

9) Thank you for stating that "Reagents generated in this study may be requested from michelle.yu-hao.lee@emory.edu with a completed materials transfer agreement". It seems that the contact email address you provided is an individual email address. Please provide a general contact email address for the data requests, so readers can request access in perpetuity. If a general email is not available, please provide a link to a website where readers can obtain access to data.

10) Please ensure that the funders and grant numbers match between the Financial Disclosure field and the Funding Information tab in your submission form. Note that the funders must be provided in the same order in both places as well.

Note: If the reviewer comments include a recommendation to cite specific previously published works, please review and evaluate these publications to determine whether they are relevant and should be cited. There is no requirement to cite these works unless the editor has indicated otherwise.

**Reviewers' Comments:**

Reviewer's Responses to Questions

**Part I - Summary**

Please use this section to discuss strengths/weaknesses of study, novelty/significance, general execution and scholarship.

Reviewer #1: The manuscript by Edwards et al. describes the adaptation of LIBRA-seq to characterize antibody responses in rhesus macaques. The authors developed DNA-barcoded BG505 SOSIP probes for sorting antigen-specific B cells, 10X capture, and single-cell RNA sequencing. They validated this approach using engineered cell lines and then applied it to identify antigen-specific memory B cells from six vaccinated animals. This approach recovered four previously identified neutralizing antibodies from animal RUp16, while identifying six additional neutralizing clones across the cohort. High-throughput analysis revealed patterns of clonal expansion and shared genetic features across animals. The LIBRA-seq scores appear useful for prioritizing antibody candidates for functional testing. However, the manuscript lacks a direct comparison between LIBRA-seq and conventional antibody cloning methods, as well as a discussion of LIBRA-seq’s limitations. Most notably, while the authors assert that LIBRA-seq "accelerates" antibody discovery, they provide no quantitative comparisons of cost, time, or workflow efficiency relative to conventional approaches. Additionally, the identification of a potent neutralizing antibody (RUp16_158_TriplePositive) that would have been excluded based on their filtering strategy, which requires biotin-bait negativity, raises concerns about the analytical pipeline that are inadequately addressed. With revisions addressing these comparisons and technical limitations, this work would represent a valuable methodological contribution to preclinical non-human primate studies.

Reviewer #2: In this study, Edwards et al apply the LIBRA-seq platform to memory B cells from rhesus macaques that received an experimental HIV vaccine, enabling immunogen-specific between IgH/L sequences to be linked with their antigen specificity. They report findings from a total of 6 vaccinated animals, one of which was previously described to have mounted a particularly potent response. The authors validate the performance of their approach by analyzing the recovery of previously-characterized clones, and also by expressing newly-identified antibodies, which together indicate good sensitivity and specificity. Overall, the study is soundly-designed, well-communicated and its conclusions are supported by the data shown.

In light of the prior studies by these authors on the same types of antibodies from the same animals, and reaching a number of the same scientific conclusions, I note that the main import of the work is as a methodological advance. However, these relevant citations are included and I think this methodological focus is frankly communicated and discussed.

Reviewer #3: Edwards, et.al., describe the adaptation and a proof-of-concept dataset demonstrating LIBRA-seq identification of binding and neutralizing antibodies targeting BG505 SOSIP in the rhesus macaque model of HIV/SIV vaccination. The team demonstrates that the adapted method is robust, reproduces prior results, and increases screening throughput for the identification and characterization of antigen-specific monoclonal antibodies. These results represent a discernable improvement over more classical, plate-based methods, and will allow more robust evaluation of humoral responses in the context of pre-clinical studies with non-human primates. This reviewer would like to commend the authors on the extremely thorough description of methodological details, which will surely encourage others to build from these findings. Overall, the paper describes a new approach that will surely increase our understanding of antibody responses during pre-clinical vaccination studies and, with relatively minor updates, should be acceptable for publication in PLoS Pathogens.

**********

**Part II – Major Issues: Key Experiments Required for Acceptance**

Please use this section to detail the key new experiments or modifications of existing experiments that should be absolutely required to validate study conclusions.

Generally, there should be no more than 3 such required experiments or major modifications for a "Major Revision" recommendation. If more than 3 experiments are necessary to validate the study conclusions, then you are encouraged to recommend "Reject".

Reviewer #1: 1. The authors assert that LIBRA-seq "accelerates" neutralizing antibody discovery in rhesus macaques. However, they provide no quantitative data to support this claim. The manuscript should include explicit comparisons of (i) hit rates for neutralizing antibody identification between LIBRA-seq and conventional approaches and (ii) estimates of the time required from sample collection to antibody characterization for each approach. Given that the authors previously characterized RUp16 using conventional methods, these comparative data should be readily available. Without such quantitative support, claims of improved efficiency cannot be substantiated.

2. The observation that 50% (2/4) of antibodies with high biotin-bait LIBRA-seq scores bound BG505 SOSIP by ELISA, with Rup16_158_TriplePositive exhibiting potent neutralizing activity, indicates that the biotin-negative filtering strategy may misclassify antigen-specific clones. The authors should quantify how many cells were excluded based on biotin-positivity across the entire cohort and determine whether these excluded cells cluster with double-positive for BG505 and negative for biotin (as observed for RUp16_158_TriplePositive and its dual-positive clonal relatives). They should also discuss the implications of these findings and recommend filtering options for future studies implementing LIBRA-seq.

Reviewer #2: (No Response)

Reviewer #3: Major Concerns:

- Antibody clonal assignments were made by identifying shared V and J genes, and shared CDR3 length, alongside CDR3 identity >70% at the nucleotide level. This seems quite a low identity threshold for a clonal grouping. Further explanation as to how and why these parameters were set for identifying members of the same clonal group would strengthen the overall narrative.

- Figure 4 and associated text demonstrate comparative analyses between the bnAbs identified by LIBRA-seq versus prior studies using more conventional methods in animal RUp16. Approximately 50% (24/48) of the identified heavy chains and 90% (44/48) of light chains agreed with prior analyses. Although the agreement on light chains is clear, the authors do not comment on the lack of overlap of heavy chains. Further discussion on whether this may represent sampling bias or due to a different time point examined would be helpful to agree with the authors conclusions that this constitutes clear orthogonal validation of the LIBRA-seq approach.

- The final Results section defines repertoire features associated with those monoclonal Abs identified by the LIBRA-seq method, including the public usage of IGHV4-79. This is a great example of how this type of method could identify new germline alleles to be targeted by rationally designed immunogens using reverse vaccinology. It was unclear whether the additional animals examined (beyond Rup16) had been characterized using more conventional methods. If the data are available, it would considerably strengthen the overall conclusions to perform similar analyses with “conventional” data. In the animals (or, if not available, if just in Rup16) did the authors see the use of IGHV4-79? Did they see high levels of SHM? In general, these types of broader analyses between standard and LIBRA-seq methods would assist in alleviating any concerns regarding LIBRA-seq-specific bias in repertoire capture and characterization.

- This adapted LIBRA-seq method identified both binding and neutralizing antibodies. The most important concern for the vaccine field is to focus on the identification of bnAbs, as binding antibodies are developed at high titers in most infected individuals. It would be beneficial for the authors to discuss possible future improvements to the assays or analyses parameters that may enhance the discernment between binding and neutralizing antibody identification.

- Data sharing remains unclear, as GEO accessions are “TBD” and git-hub documentation “will be made available”. These availabilities need to be guaranteed and made available to reviewers prior to acceptance.

**********

**Part III – Minor Issues: Editorial and Data Presentation Modifications**

Please use this section for editorial suggestions as well as relatively minor modifications of existing data that would enhance clarity.

Reviewer #1: 1. The authors should include more discussion of the technical challenges of implementing LIBRA-seq and the approach's limitations, including practical considerations for those considering LIBRA-seq, the technical expertise required, and the computational resources. This information will help readers assess the feasibility of implementing LIBRA-seq.

2. HVV should be defined the first time it is used in the manuscript.

Reviewer #2: – Given that only a single antigen is being used, the contribution of the DNA-barcode on the antigen probes is somewhat unclear: almost all of the discrimination appears to have been done by the fluorescent sorting (eg see Fig 3). How much would the sensitivity / specificity of recovering immunogen-specific IgH/Ls actually have dropped if there was no DNA barcode quantification available? In addition to excluding what appear to be a small number of fluorescent false positives, perhaps the main value of the barcode is allowing a quantitative estimation of binding signal strength / reliability? In that vein, it would have been helpful to see a more thorough and quantitative analysis of sensitivity / specific across the LSS range, and/or some further discussion on this point.

– The titration in Figure 2 would have benefitted from replicates, or an explanation of why none were performed.

– Fig 6: "D" missing from legend.

Reviewer #3: Minor concerns:

In general, the paper was well written and clear. There were a number of typos and minor comments listed here to assist in the readability of the manuscript once it is published:

- Line 269: there are words missing at the end of this sentence, “…0.022-0.039 in IgK, and (0.025-0.044).

- Line 283: I believe that this wording should be adjusted to, “high resolution, higher throughput manner…” to more accurately represent the data presented.

- Lines 289-291, “In addition to recovering the previously identified neutralizing clones, we were able to identify shared gene usage and clonotypes across multiple variants.” While not untrue, this statement felt like a bit of an overreach, as the authors did not identify an exact overlap between methods. This reviewer suggests rewording to “…was able to identify partial shared gene usage…” to better temper the results.

- Lines 428 and 440. Please use the correct symbol for degrees.

- Line 447, this sentence starts with, “(AMQAX1000) Appropriate volume…” Clearly something was lost in editing, please correct.

- Line 452-453, Poor grammar and articles are missing; please review and correct.

- Throughout manuscript – the Ramos cell line is referred to as “ramos” and “Ramos”. Please decide regarding capitalization and be consistent throughout the manuscript.

- Lines 533-534, it is unclear how and what was “combined at 1 to 1 overnight…”. Were streptavidin-AF and oligonucleotide mixed at 1:1 based on vol:vol, or mass:mass? Clarify methods here to increase reproducibility.

- Multiple times the term “x” was used as shorthand for replicates. Please expand. For example, line 551, “Splenocytes were washed 3x with FACS buffer…” should be “Splenocytes were washed three times with FACS buffer...”.

- Single-cell RNA-seq using 10X Genomics equipment and reagents was critical for this study. However, these supplies are referred to repeatedly as either “10x” or “10X”. Please use the proper “10X Genomics” and be consistent throughout draft.

- Lines 626-636, this section appears to be completely duplicated from the prior section (lines 590-601) and can be removed.

- The “Libra-seq Scoring” methods section (lines 637-649) also seems almost word-for-word duplicated from an earlier methods section (lines 602-612). Please rework to limit redundancy.

**********

PLOS authors have the option to publish the peer review history of their article (what does this mean?). If published, this will include your full peer review and any attached files.

If you choose “no”, your identity will remain anonymous but your review may still be made public.

**Do you want your identity to be public for this peer review?**  For information about this choice, including consent withdrawal, please see our Privacy Policy.

Reviewer #1: No

Reviewer #2: No

Reviewer #3: No

[NOTE: If reviewer comments were submitted as an attachment file, they will be attached to this email and accessible via the submission site. Please log into your account, locate the manuscript record, and check for the action link "View Attachments". If this link does not appear, there are no attachment files.]

**Figure resubmission:**

-->While revising your submission, we strongly recommend that you use PLOS’s NAAS tool (https://ngplosjournals.pagemajik.ai/artanalysis) to test your figure files. NAAS can convert your figure files to the TIFF file type and meet basic requirements (such as print size, resolution), or provide you with a report on issues that do not meet our requirements and that NAAS cannot fix.

After uploading your figures to PLOS’s NAAS tool - https://ngplosjournals.pagemajik.ai/artanalysis, NAAS will process the files provided and display the results in the "Uploaded Files" section of the page as the processing is complete. If the uploaded figures meet our requirements (or NAAS is able to fix the files to meet our requirements), the figure will be marked as "fixed" above. If NAAS is unable to fix the files, a red "failed" label will appear above. When NAAS has confirmed that the figure files meet our requirements, please download the file via the download option, and include these NAAS processed figure files when submitting your revised manuscript.

**Reproducibility:**

To enhance the reproducibility of your results, we recommend that authors of applicable studies deposit laboratory protocols in protocols.io, where a protocol can be assigned its own identifier (DOI) such that it can be cited independently in the future. Additionally, PLOS ONE offers an option to publish peer-reviewed clinical study protocols. Read more information on sharing protocols at https://plos.org/protocols?utm_medium=editorial-email&utm_source=authorletters&utm_campaign=protocols

# Author comment: Author response to Decision Letter 1

**DOI:** 10.1371/journal.ppat.1013831.r002

18 May 2026

Attachment Submitted filename: Response to Reviewers.docx

# Peer review: Decision Letter 1

**DOI:** 10.1371/journal.ppat.1013831.r003

**Authors:** Susan R. Ross, David T. Evans

2 Jun 2026

Dear Dr. Bosinger,

We are pleased to inform you that your manuscript 'Non-human primate LIBRA-Seq accelerates neutralizing antibody discovery in RM vaccinated against HIV-1' has been provisionally accepted for publication in PLOS Pathogens.

Before your manuscript can be formally accepted you will need to complete some formatting changes, which you will receive in a follow up email. A member of our team will be in touch with a set of requests.

Please note that your manuscript will not be scheduled for publication until you have made the required changes, so a swift response is appreciated.

IMPORTANT: The editorial review process is now complete. PLOS will only permit corrections to spelling, formatting or significant scientific errors from this point onwards. Requests for major changes, or any which affect the scientific understanding of your work, will cause delays to the publication date of your manuscript.

Should you, your institution's press office or the journal office choose to press release your paper, you will automatically be opted out of early publication. We ask that you notify us now if you or your institution is planning to press release the article. All press must be co-ordinated with PLOS.

Thank you again for supporting Open Access publishing; we are looking forward to publishing your work in PLOS Pathogens.

Best regards,

David T. Evans

Academic Editor

PLOS Pathogens

Susan Ross

Section Editor

PLOS Pathogens

Sumita Bhaduri-McIntosh

Editor-in-Chief

PLOS Pathogens

orcid.org/0000-0003-2946-9497

Michael Malim

Editor-in-Chief

PLOS Pathogens

orcid.org/0000-0002-7699-2064

***********************************************************

Reviewer Comments (if any, and for reference):

Reviewer's Responses to Questions

**Part I - Summary**

Please use this section to discuss strengths/weaknesses of study, novelty/significance, general execution and scholarship.

Reviewer #1: The authors have addressed the major concerns raised in the initial review. The addition of Table 2 provides a direct comparison between LIBRA-Seq and their conventional plate-based cloning method, supporting the claim that LIBRA-Seq reduces the time and labor required for antibody discovery. The overview of the technical requirements and limitations of the NHP-adapted LIBRA-Seq workflow is helpful for readers new to the technique. The reanalysis of triple-negative cells clarifies their relationships with antigen-specific lineages, and the Discussion recommends treating positive-bait signals as a quality-control measure rather than for strict exclusion. With minor editorial corrections detailed below, the adaptation of LIBRA-Seq to nonhuman primate models provides an additional tool for evaluating antigen-specific B cells in preclinical studies.

Reviewer #2: The authors have satisfactorily addressed my concerns.

Reviewer #3: The authors carefully considered the feedback provided by all three reviewers, made appropriate changes to strengthen the manuscript, and more thoroughly describe the implementation and limitations of the LIBRA-seq methods. This reviewer believes the revised manuscript to now be suitable for publication.

**********

**Part II – Major Issues: Key Experiments Required for Acceptance**

Please use this section to detail the key new experiments or modifications of existing experiments that should be absolutely required to validate study conclusions.

Generally, there should be no more than 3 such required experiments or major modifications for a "Major Revision" recommendation. If more than 3 experiments are necessary to validate the study conclusions, then you are encouraged to recommend "Reject".

Reviewer #1: (No Response)

Reviewer #2: (No Response)

Reviewer #3: N/A

**********

**Part III – Minor Issues: Editorial and Data Presentation Modifications**

Please use this section for editorial suggestions as well as relatively minor modifications of existing data that would enhance clarity.

Reviewer #1: 1. The number of dual-positive antibodies tested by ELISA is inconsistent across the text, Figure 5, and Table 2. Line 238 states that “All 30 antibodies with LIBRA-Seq scores denoting a high specificity for BG505 SOSIP exhibited binding via ELISA,” and Table 2 lists 30 antibodies as expressed. However, Figure 5B shows only 29 antibodies (with only four antibodies from animal Rlr15), and two antibodies from animal Rys15 that do not bind appreciably to BG505 SOSIP. The “Binding Abs (by ELISA)” row of Table 2 reports “27/29 93% overall,” which is consistent with Figure 5B but not the statement on line 238. The authors should reconcile these discrepancies and update the text to accurately reflect the number of samples run and their binding to BG505.

2. The reference to Suppl. Fig. S4A on line 226 appears incorrect. Based on the context (sorting of biotin-negative memory B cells from the five additional vaccinated animals), this citation should likely be Suppl. Fig. S5A. The authors should verify the correct reference to the supplementary figure.

Reviewer #2: (No Response)

Reviewer #3: N/A

**********

PLOS authors have the option to publish the peer review history of their article (what does this mean?). If published, this will include your full peer review and any attached files.

If you choose “no”, your identity will remain anonymous but your review may still be made public.

**Do you want your identity to be public for this peer review?**  For information about this choice, including consent withdrawal, please see our Privacy Policy.

Reviewer #1: No

Reviewer #2: **Yes:** John A Altin

Reviewer #3: No

# Editor report: Acceptance letter

**DOI:** 10.1371/journal.ppat.1013831.r004

**Authors:** Susan R. Ross, David T. Evans

Dear Dr. Bosinger,

We are delighted to inform you that your manuscript, "Non-human primate LIBRA-Seq accelerates neutralizing antibody discovery in RM vaccinated against HIV-1," has been formally accepted for publication in PLOS Pathogens.

We have now passed your article onto the PLOS Production Department who will complete the rest of the pre-publication process. All authors will receive a confirmation email upon publication.

The corresponding author will soon be receiving a typeset proof for review, to ensure errors have not been introduced during production. Please review the PDF proof of your manuscript carefully, as this is the last chance to correct any scientific or type-setting errors. Please note that major changes, or those which affect the scientific understanding of the work, will likely cause delays to the publication date of your manuscript. Note: Proofs for Front Matter articles (Pearls, Reviews, Opinions, etc...) are generated on a different schedule and may not be made available as quickly.

Soon after your final files are uploaded, the early version of your manuscript, if you opted to have an early version of your article, will be published online. The date of the early version will be your article's publication date. The final article will be published to the same URL, and all versions of the paper will be accessible to readers.

For Research Articles, you will receive an invoice from PLOS for your publication fee after your manuscript has reached the completed accept phase. If you receive an email requesting payment before acceptance or for any other service, this may be a phishing scheme. Learn how to identify phishing emails and protect your accounts at https://explore.plos.org/phishing.

Thank you again for supporting open-access publishing; we are looking forward to publishing your work in PLOS Pathogens.

Best regards,

Sumita Bhaduri-McIntosh

Editor-in-Chief

PLOS Pathogens

orcid.org/0000-0003-2946-9497

Michael Malim

Editor-in-Chief

PLOS Pathogens

orcid.org/0000-0002-7699-2064
